# Imaging of groin pain in athletes: patterns of injury at MRI and gender differences therein

**DOI:** 10.1007/s11845-022-03126-3

**Published:** 2022-08-16

**Authors:** John P. Hynes, Meadhbh O’Flaherty, David Glynn, Sarah Eustace, Eoin C. Kavanagh

**Affiliations:** 1 Department of Radiology, National Orthopaedic Hospital of Ireland, Cappagh, Dublin, Ireland; 2 Mater Misericordiae University Hospital, Dublin, Ireland

**Keywords:** Athletic groin pain, Athletic imaging, Femoroacetabular impingement, Groin pain imaging, Sports imaging, Sports medicine

## Abstract

**Aim:**

The purpose of our study was to review a large cohort of athletes of all levels presenting with groin pain who underwent investigation with MRI and to determine what the commonest patterns of injury were. We aimed to explore whether particular findings were commonly found in association and whether measurable gender differences exist in the incidence of specific injuries.

**Materials and methods:**

Imaging records were reviewed to identify MRI studies of the pelvis performed for the investigation of groin pain in patients who were active in sports/athletic pursuits. Findings were classified and recorded as follows: injury to the common rectus abdominis/adductor longus origin, injury to the short adductor muscles, pubic bone oedema, pubic symphysis degenerative changes, hip joint injury and ‘other’. The prevalence of specific injuries in female athletes compared to males was analysed using relative risk ratios.

**Results:**

A total of 470 athletes underwent MRI for the investigation of groin pain during the study period. Forty-six were female, and 424 were male. Female athletes were significantly less likely to have rectus abdominis-adductor longus (RR = 0.31, *p* = .017), short adductor (RR = 0.14, *p* = .005) or hip (RR = 0.41, *p* = .003) injuries. Pubic bone degenerative changes were much more common in female athletes (RR = 7.37, *p* = .002).

**Conclusion:**

Significant gender differences exist in the frequency with which specific injuries are observed. Female athletes are also significantly underrepresented; this is likely a multifactorial phenomenon; however, the possibility of unconscious referrer bias must be considered.

**Supplementary Information:**

The online version contains supplementary material available at 10.1007/s11845-022-03126-3.

## Introduction

Groin pain in athletes of all abilities, from elite competitors to ‘weekend warriors’, is common and may be very debilitating [1]. It occurs across virtually all sports; however, it is thought to be more common in multidirectional sports and those which involve kicking [2]. Time lost from sport due to groin pain can be significant, which has a particular impact in the professional sphere where careers may be compromised [3].

Groin pain is notoriously difficult to accurately diagnose and manage, posing a dilemma for the sports physician. Injuries occurring across a large anatomical region involving complex interrelating structures may contribute to groin pain, and frequently pathologies may co-exist [4]. Magnetic resonance imaging (MRI) is the mainstay of investigation in groin pain due to the unparalleled soft tissue definition it affords and may be crucial to accurate diagnosis, affording correct and timely intervention [5].

The purpose of our study was to review a large number of MRI studies performed to investigate groin pain in athletes of all levels and to determine what the commonest findings and potential causes for pain were. We aimed to explore whether particular findings were commonly found in association and whether measurable gender differences exist in the incidence of specific injuries.

## Anatomy

The groin lacks formal anatomical definition and boundaries; for the purposes of this discussion, it may be considered the area encompassing the pubic symphysis, the proximal portion of the adductor compartment of the thighs and the bilateral inguinal regions. Pathologies occurring remote to this area may also contribute to and manifest as groin pain, however, in particular injuries to the hip joint [6].

The pubic bodies bilaterally are joined by a fibrocartilaginous articular disc, constituting a non-synovial symphyseal joint which is encircled by a fibrous capsule. The central articular disc contains a small, physiological fluid-filled cleft. The pubic symphysis helps to afford stability to the pelvis [7].

The rectus abdominis muscle functions as an important stabiliser of the abdominal wall. It originates from the pubic symphysis and adjacent pubic crest, and medial and lateral heads may be distinguished bilaterally. The medial and lateral heads taper to an aponeurosis, which originates at the anterior pubis. Importantly, this aponeurosis is continuous with the adjacent origin of the adductor longus tendons, forming a common structure which is sometimes referred to as the pre-pubic aponeurotic complex or PPAC. The PPAC may be identified on sagittal MR images adherent to the pubis anteriorly [8].

The adductor longus muscle helps to stabilise the pelvis during gait and adducts the thigh. It passes from its common origin with the rectus abdominis to a relatively broad insertion onto the middle third of the femur. The adductor brevis and gracilis muscles originate slightly further laterally and posteriorly, from the inferior pubic body and pubic ligament. These tendons are variably fused with one another. The pectineus muscle originates from the superior pubic crest, lying immediately lateral to the lateral head of rectus abdominis.

While the anatomy of the groin region and pubic symphysis is complex, a thorough understanding of the entities involved and their structural relationships is integral to understanding the mechanism of different types of groin injuries and accurately interpreting the appearances at MRI.

The hip joint is a highly mobile ball and socket synovial articulation between the femoral head and the spherical acetabulum. The acetabular labrum is a fibrocartilaginous structure which acts to increase the depth and stability of the joint. A fibrous capsule surrounds the hip joint attaching to the margins of the labrum, the transverse ligament at the acetabular notch and the intertrochanteric line. The hip may be subject to a variety of injuries in the athletic population and must always be considered in the evaluation of groin pain [9].

## Methods

Institutional ethical approval was sought and obtained for a retrospective review of imaging records. Our institution is a national specialist centre for orthopaedics and sports medicine.

Imaging records were reviewed to identify MRI studies of the pelvis performed for the investigation of groin pain in patients who were active in sports or athletic pursuits. Studies were excluded if there were multiple sites of pain (i.e. ‘hip’ and ‘groin’ pain). MRI was performed using a 3-T unit (Achieva TX, Philips Healthcare) and a 16-element phased-array receive-only coil. The following sequences were acquired: coronal STIR (inversion time [TI], 200 ms; TR/TE, 5024/60; FOV, 375 × 375 mm; slice thickness, 4 mm; matrix, 384 × 300), coronal T1-weighted (TR/TE, 569/20; FOV, 360 × 375 mm; slice thickness, 3 mm; matrix, 492 × 462), axial T2-weighted (TR/TE, 5554/130; FOV, 375 × 375 mm; slice thickness, 3.5 mm; matrix, 512 × 397) and sagittal STIR (TI, 200 ms; TR/TE, 5024/60; FOV, 265 × 207 mm; slice thickness, 4 mm; matrix, 272 × 168).

**Fig. 1:**
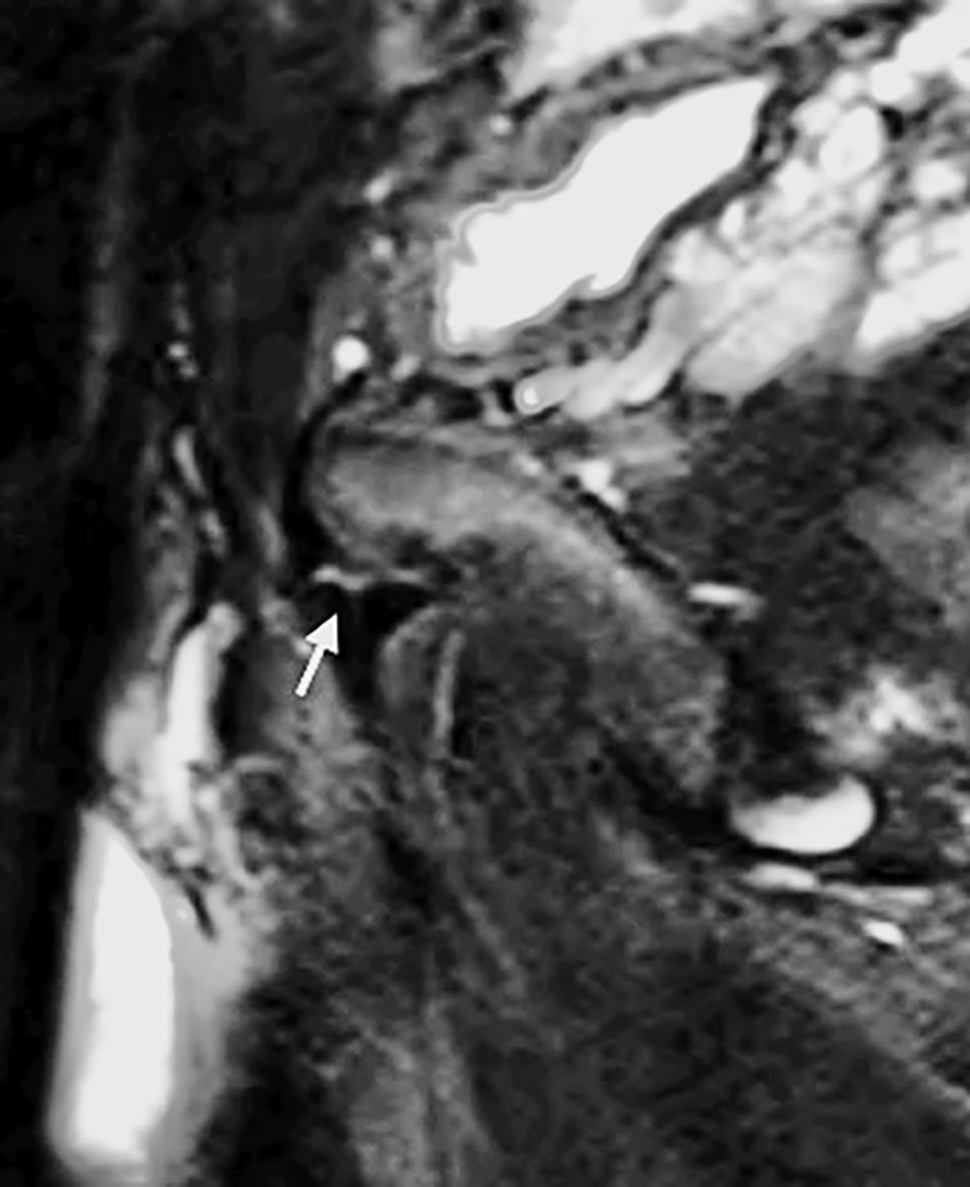
Sagittal STIR image of the pelvis in a 32-year-old male athlete demonstrating a tear at the rectus abdominis/adductor longus insertion (white arrow)

**Fig. 2:**
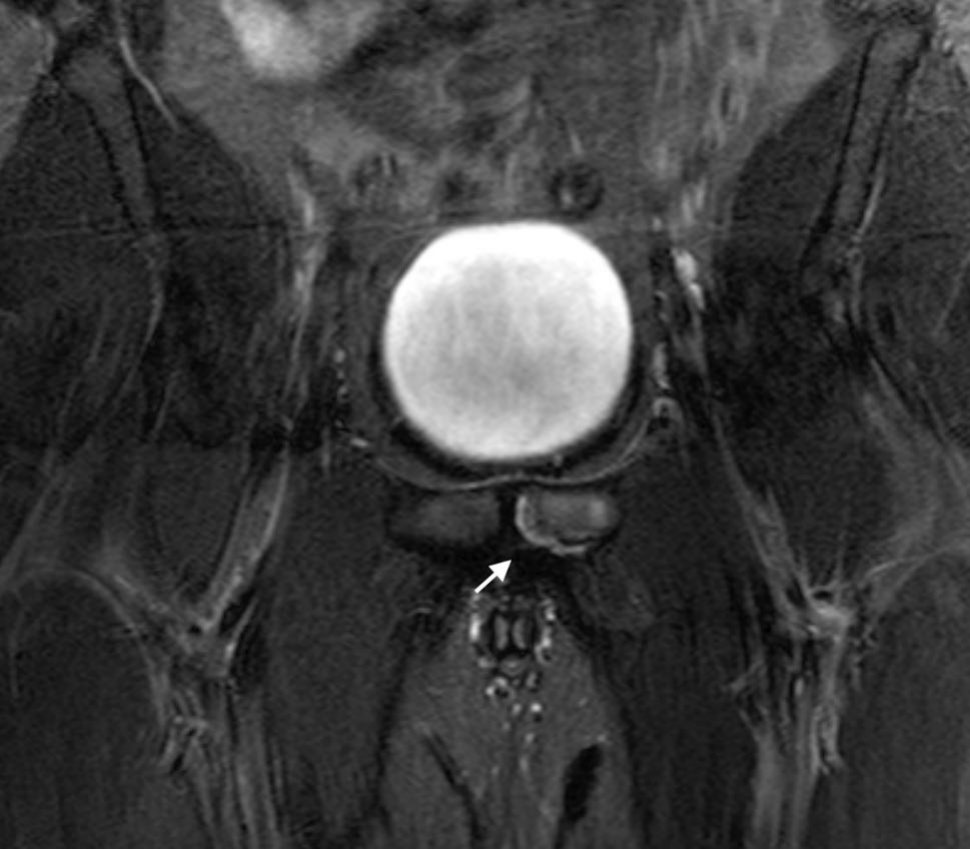
Coronal STIR image of the pelvis in a 26-year-old male athlete demonstrating linear signal intensity along the inferior margin of the superior pubic ramus (white arrow)—this is the superior cleft sign

**Fig. 3:**
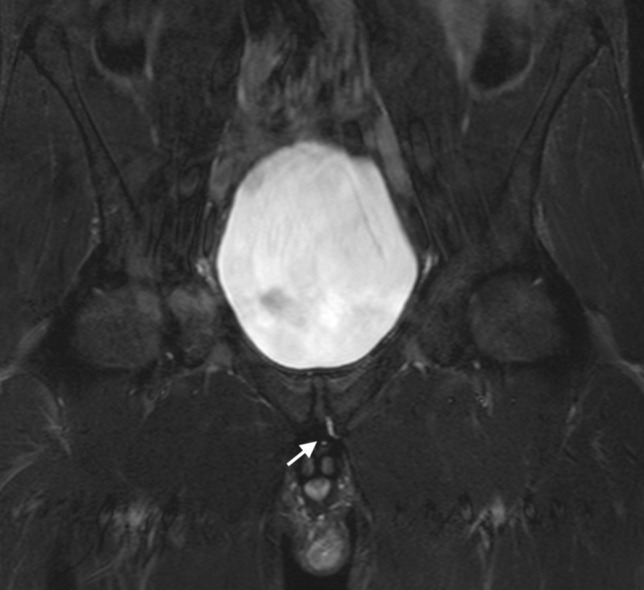
Coronal STIR image of the pelvis in a 29-year-old male athlete demonstrating linear signal intensity along the inferior margin of the inferior pubic ramus (white arrow)—this is the secondary cleft sign

**Fig. 4:**
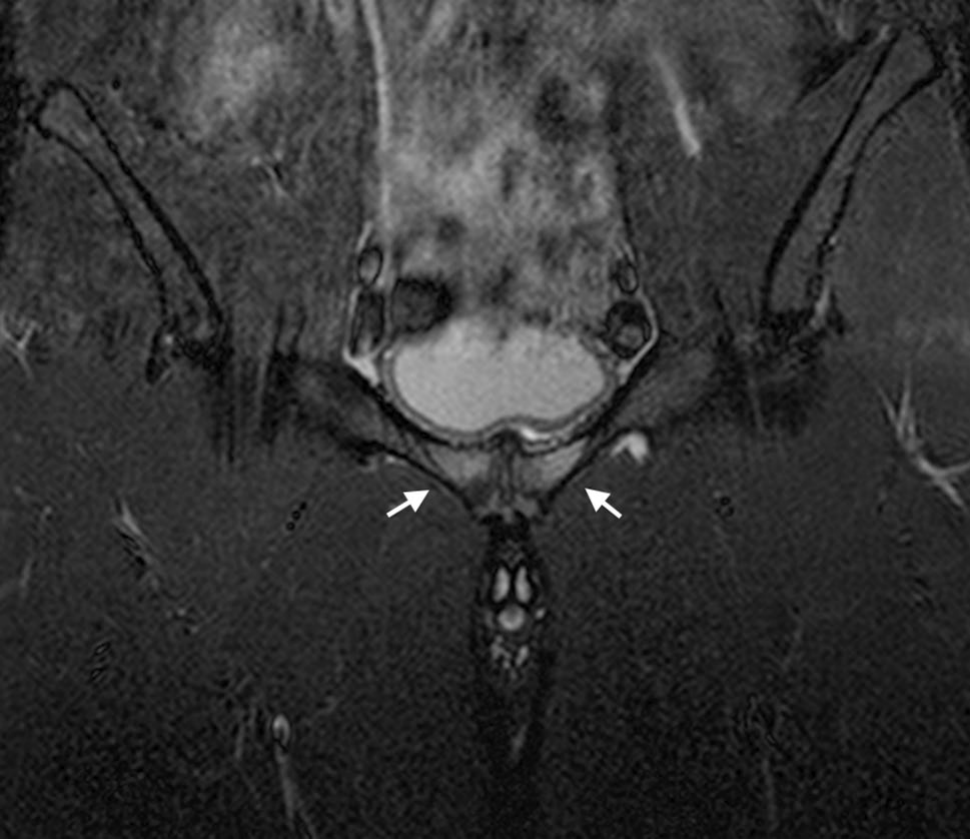
Coronal STIR image of the pelvis in a 21-year-old male athlete demonstrating bone marrow oedema in the pubic bones bilaterally (arrows) spanning the pubic symphysis

**Fig. 5:**
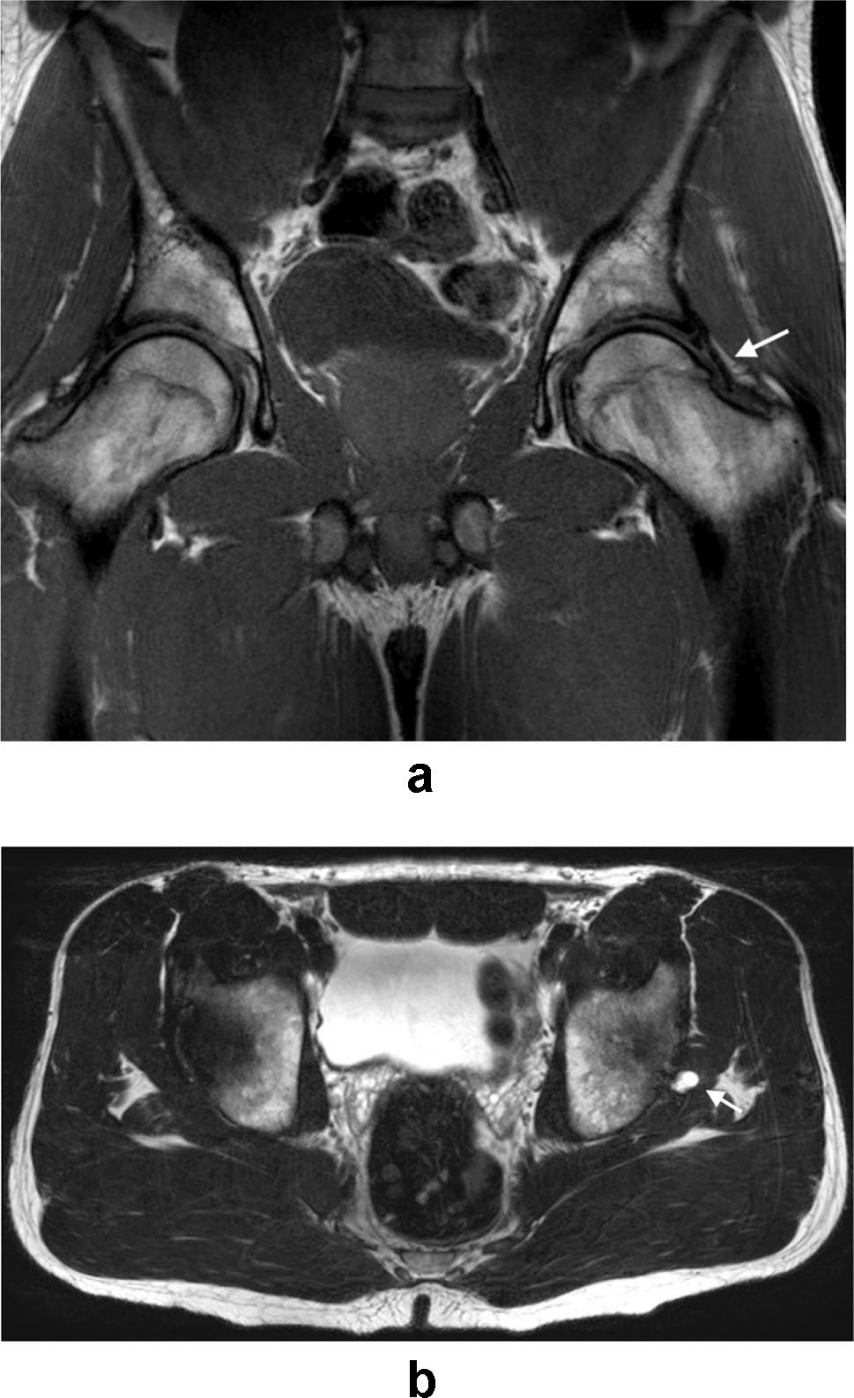
**A** Coronal T1-weighted image of the pelvis in a 34-year-old male athlete demonstrating subtle protuberance at the femoral head-neck junction (arrow) in keeping with Cam-type impingement morphology. There is associated early degenerative partial-thickness chondrosis at the hip joints. **B** Axial T2-weighted image in the same patient demonstrating a small left paralabral cyst (arrow) consistent with an underlying labral tear

Following the identification of relevant imaging studies, images were reviewed by two musculoskeletal radiologists with 21 and 17 years respectively of consultant experience in consensus read. Findings thought to be relevant to groin pain were classified and recorded as follows:

- Injury to the common rectus abdominis/adductor longus origin (Fig. 1).
- Injury to the short adductor muscles or their attachments (gracilis, adductor brevis and pectineus muscles [10]).
- Muscular attachment injuries were recorded where there was partial or full thickness tearing, intramuscular fluid signal intensity or fluid signal intensity appreciated around a myotendinous junction. The ‘superior cleft’ sign (Fig. 2) and ‘secondary cleft’ sign (Fig. 3) were accepted as markers of injury to the rectus abdominis/adductor longus attachment and short adductors attachments, respectively.
- Pubic bone oedema (defined as bone oedema spanning the pubic symphysis and remote from muscular attachments, see Fig. 4).
- Pubic symphysis degenerative changes (joint space narrowing, irregularity of the articular surfaces and osteophytosis).
- Injury to the hip joint (findings included subchondral oedema, degenerative changes with chondrosis, labral oedema or paralabral cyst formation [see Fig. 5]. Impingement morphology alone in the absence of secondary labral derangement was not included).
- ‘Other’ findings.

The number of patients with each of these findings was recorded. Where more than one finding was present, the combination of injuries was also recorded. The prevalence of specific injuries in female athletes compared to males was analysed using relative risk ratios. Approval from the Institutional Review Board was obtained, and in keeping with the policies for a retrospective review, informed consent was not required.

All authors contributed to the overall concept and design of the study. JH, MOF, DG and SE performed data collection, data processing, collation and preparation of images for publication. JH and MOF drafted and revised the manuscript. EK supervised the writing, provided critical feedback and lead the overall direction and planning.

## Results

A total of 470 athletes underwent MRI for the investigation of groin pain during the study period. Forty-six were female, and 424 were male (F:M;1:9.2). The age range was 16–49, with an average age of 24.9. Female athletes were significantly older than male athletes (29.5 vs 24.6, *p* < 0.05). In 119 of the 470 athletes, the MRI scan was normal with no cause for groin pain demonstrated (25.3%). A total of 464 findings were recorded in the remaining 349 patients. Two hundred fifty-four athletes (54.0%) had 1 finding, 73 athletes (15.5%) had 2 findings, 20 athletes (4.3%) had 3 findings, and 1 patient had four findings.

**Fig. 6:**
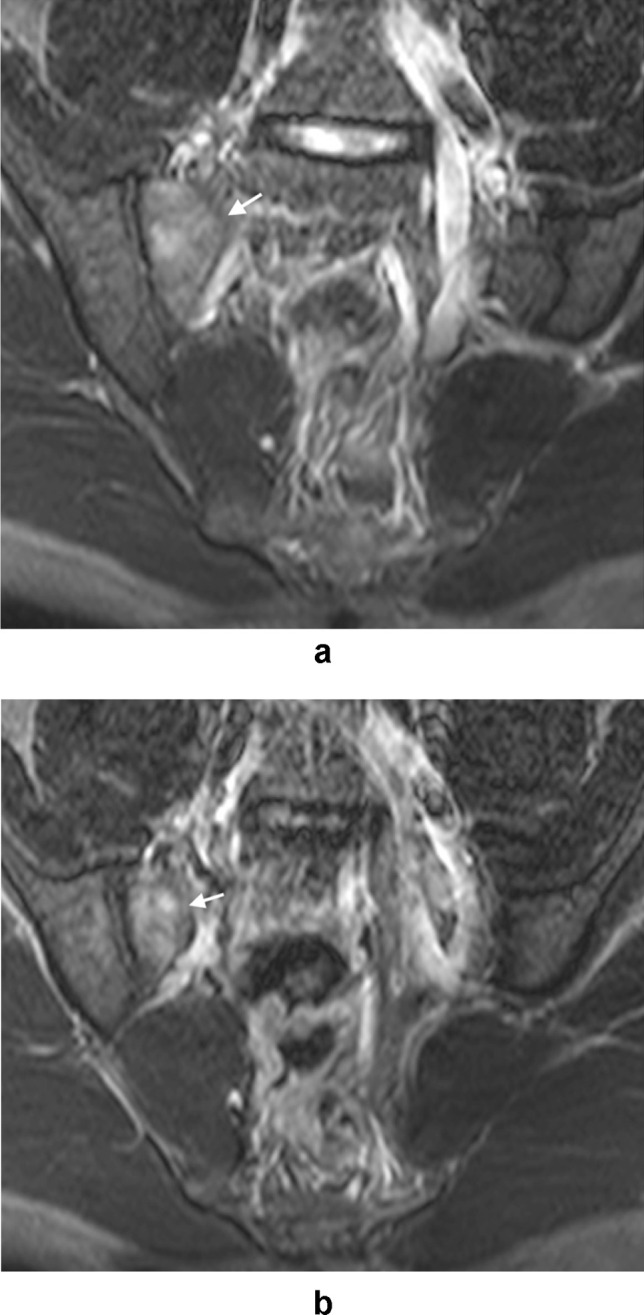
**A** and **B** Axial STIR images of the pelvis in a 28-year-old female athlete with right groin pain demonstrating oedema along the anterior margins of the right sacroiliac joint (arrows) in keeping with sacroiliitis

**Fig. 7:**
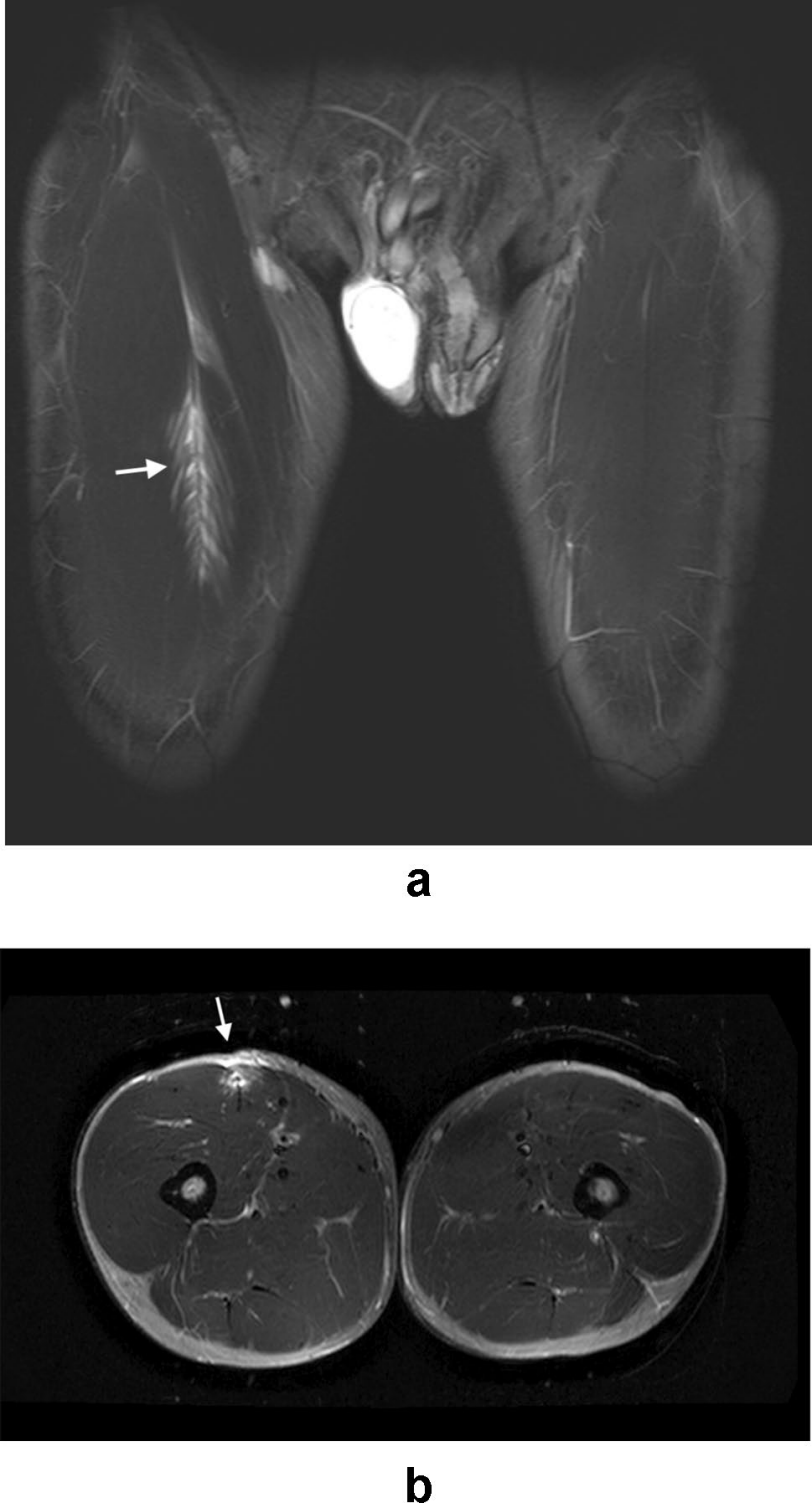
**A** (coronal STIR) and **B** (axial STIR) images in a 22-year-old male athlete with right groin pain demonstrate a tear of the right rectus femoris muscle

The commonest finding at MRI was injury to the short adductor muscles which was recorded in 133 athletes (28.3%) followed by injury to the joint rectus abdominis-adductor longus attachment in 125 athletes (26.6%). Ninety-five athletes had pubic bone oedema (20.2%), while degenerative changes at the pubic symphysis were recorded in only 9 patients (1.9%). Seventy-one athletes (15.1%) had abnormalities at the hip joint, and 37 athletes (7.9%) had relevant findings classified as ‘other’. A comprehensive breakdown of findings is provided in Table 1, and ‘other’ findings are described in Table 2 (see also Figs. 6 and 7).

**Table 1:** Frequency of categories of injury at MRI

Finding	Rectus abdominis-adductor longus	Short adductor	Pubic bone oedema	Pubic degenerative changes	Hip	‘Other’	Normal/no findings
No. of athletes	125	133	95	9	71	37	119
%	26.6	28.3	20.2	1.9	15.1	7.9	25.3

^*^Sum of percentages is > 100% as more than one finding may occur in each patient

**Table 2:** Gender differences in the incidence of injury categories

	Risk ratio for female athletes (versus male)	*p*	95% CI
Finding			
Rectus abdominis-adductor longus	0.31	.0166	0.12–0.81
Short adductor	0.14	.0048	0.03–0.55
Pubic bone oedema	0.73	.3896	0.36–1.39
Pubic degenerative changes	7.37	.0022	2.05–26.48
Hip	0.41	.1139	0.13–1.24
‘Other’	1.78	.1662	0.79–4.05
Normal/no findings	1.79	.003	1.21–2.63

A total of 94 athletes (92 male, 2 female) had 2 or more injuries at MRI. The commonest combination of patterns of findings was injury to the short adductors and the rectus abdominis-adductor longus attachment, which was recorded in 21 athletes. Fifteen athletes had short adductor, rectus abdominis/adductor longus injuries and pubic bone oedema. A further 15 had pubic bone oedema in combination with short adductor injuries. Table 3 provides further details on injury combinations.

**Fig. 8:**
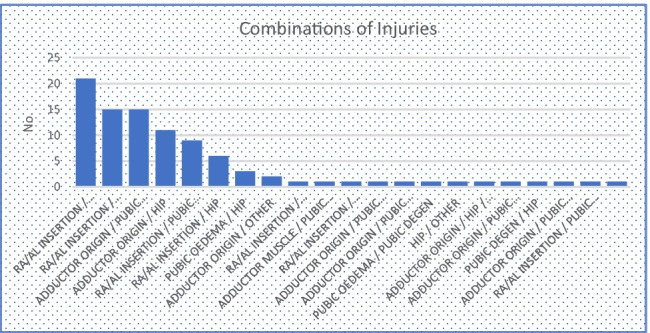
Combinations of injuries

**Table 3:** ‘Other’ findings

Finding	No. of athletes
Rectus femoris tear	6
Adductor magnus tear	4
Hamstring origin tear	3
Femoral stress fracture	3
Sacro-ileitis	6
Unilateral	3
Bilateral	3
Piriformis tear	2
Obturator externus tear	2
Gluteus medius tear	2
Varicoele	2
Sartorius tear	1
Obturator internus and externus tears	1
Vastus lateralis tear	1
Myositis ossificans	1
Prostatitis	1
Pelvic congestion syndrome	1

Significant gender differences exist in the frequency with which specific injuries are observed. Pubic bone degenerative changes were much more common in female athletes (RR = 7.37, *p* = 0.002), and the mean age of female athletes in the study was higher (29.5) than that of males (24.6) which may be a contributory factor. Female athletes were also significantly less likely to have rectus abdominis-adductor longus, short adductor or hip injuries. Females were less likely to have pubic bone oedema and more likely to have ‘other’ findings; however, these trends did not reach statistical significance. 41.3% of female athletes referred for MRI for investigation of groin pain did not have any relevant findings in comparison to 20.9% of male athletes (RR = 1.79, *p* = 0.003). Fig. 8 provides further analysis of gender differences in the findings.

## Discussion

Our results demonstrate significant gender-based differences in both referral patterns and injury mechanisms in athletes with groin pain. The ratio of female to male athletes referred during the study period was 1:9.2. This is a large discrepancy when compared to gender differences in participation in sport and exercise in Ireland, with the gender gap being a mere 3% in 2019 (48% of men and 45% of women regularly participating) [11]. Similar large studies of imaging for athletic groin pain internationally demonstrate similar discrepancies, for example, the work of Zoga et al. found a ratio of 1:19.1 [19], suggesting that this phenomenon is not an isolated one.

A systematic review of groin injuries in elite sports found that men had a significantly greater incidence of groin injury than women, and the disparity in referral volume may partly reflect a higher burden of these injuries in male athletes [2]. Several anatomical differences have been suggested as possible explanations for this. The wider female pelvis produces a more oblique angle of action for the short adductors in females, which may reduce the tractional force exerted at their origins [12]. This may account for reduced insertional muscular tearing in females. There is also evidence that Cam-type impingement morphology of the hip joint (whereby there is excess bone formation at the anterolateral aspect of the femoral head-neck junction) occurs more frequently in males [13] which may predispose male athletes to labral derangements, manifesting as groin pain.

An additional factor to consider is the potential for unconscious gender bias on the part of referrers. Gender disparities have previously been demonstrated in a variety of specialties. Despite osteoarthritis being more prevalent in women, they are less likely to be offered or to undergo joint arthroplasty [14, 15]. Women were also less likely to be referred for imaging for chronic wrist pain [16]. The exact circumstances regarding the decision to refer an individual patient for imaging are of course impossible to ascertain; however, the potential for systemic implicit bias must be acknowledged. Only by being aware of gender disparity in imaging utilisation can we hope to achieve more equitable standards in the future.

Historically, the diagnosis of groin pain has posed a significant dilemma for the sports physician. This is multifactorial, in that the symptoms can be subtle and difficult to localise, the anatomy is complex—as outlined in the introduction—involving multiple interrelated structures, and pain may be referred from other regions rather than originating in the groin. These factors have likely contributed to the often confusing and imprecise nomenclature used in the description of athletic groin pain; for example ‘Gilmore’s groin’, ‘sportsman’s hernia’ and ‘athletic pubalgia’[17]. Clinical assessment and examination remains critical to the evaluation of athletic groin pain, and the Doha consensus statement on terminology in athletic groin pain sought to bring much needed clarity, whereby defined clinical entities were established—adductor-related, iliopsoas-related, inguinal-related and pubic-related groin pain [18]. The statement also allowed for hip-related groin pain and ‘other’ causes of groin pain in athletes. We contend that the incorporation of MRI can add significant additional accuracy to this approach via the precise localisation of abnormal findings. The categories we assigned to the findings at MRI correspond closely to the entities described in the Doha statement. It is worth noting that a typical MRI protocol for the investigation of groin pain does not include dedicated arthrographic or axial oblique hip imaging, and therefore it is possible that certain pathologies involving the hip joint—particularly labral tears—may be missed and therefore may be underrepresented.

Microtearing at the rectus abdominis-adductor longus insertion was described by Zoga et al. in 2008 [19]. Classically, this is associated with the superior cleft sign, with linear signal hyperintensity seen at the inferior margin of the superior pubic ramus [20]. Injury to the rectus abdominis-adductor longus complex is thought to be closely related to the clinical entity ‘sportsman’s hernia’, or what is now known as inguinal-related groin pain, due to the close proximity to the superficial inguinal ring.

Similarly, microtearing at the attachment of the short adductors manifests at MRI as the secondary cleft sign, with signal hyperintensity extending along the inferior margin of the inferior pubic ramus [21]. Injury to the short adductors or their attachments corresponds closely to ‘adductor-related groin pain’.

‘Osteitis pubis’ was another commonly utilised term in the description of groin pain; however, there is no consistency or consensus as to its definition or whether it constitutes a radiological or clinical entity. The Doha agreement describes pubic-related groin pain [12]. We characterised imaging findings in relation to the pubic bone as pubic bone oedema or degenerative changes at the symphysis. Our definition of pubic bone oedema was based on that of Zoga et al., as oedema spanning the symphysis and extending anteriorly to posteriorly [13]; focal traction oedema at muscular attachments was not included for this purpose. The incidence of pubic bone oedema in both symptomatic and asymptomatic athletic populations is highly variable in the literature (0–72% in a systematic review [22]) which is likely at least in part a function of the heterogeneity with which it is described, with an absence of recognised diagnostic criteria or grading systems [23].

Concerns have previously been raised around the potential for misinterpretation of imaging findings at MRI in the context of athletic groin pain, and activity-related changes may certainly be noted in the asymptomatic athletic population, particularly changes of pubic bone oedema [24]. However, there is little doubt that MRI—in conjunction with clinical evaluation—is critical to accurate diagnostic workup. Imaging findings relevant to groin pain are significantly more common in the symptomatic athletic population than in matched asymptomatic cohorts and demonstrate strong concordance with both physical examination and surgical findings [1, 6]. MRI is estimated to increase the probability of an individual diagnosis to 93%, from a pre-test probability of 62% based on clinical assessment alone [6].

In summary, we describe the findings at MRI in a large group of athletes referred for imaging due to groin pain. Groin pain has historically posed a diagnostic dilemma; however, the precise anatomical classification of injuries incorporated with recently updated clinical categorisation can enhance the evaluation process. Significant differences exist in the frequency with which certain injury patterns are observed in female and male athletes. Female athletes are significantly underrepresented; this is likely a multifactorial phenomenon; however, the possibility of unconscious referrer bias must be considered.

## Supplementary Information

Below is the link to the electronic supplementary material.

Supplementary file1 (PDF 191 KB)
